# Gastric lipoma: An unusual cause of the upper gastrointestinal bleeding

**DOI:** 10.1016/j.ijscr.2024.109684

**Published:** 2024-04-29

**Authors:** M. Cherif, M. Mesbahi, N. Khedhiri, Y. Benzarti, A. Ben Maamer

**Affiliations:** Department of general surgery, Habib Thameur Hospital, Tunisia

**Keywords:** Gastric lipoma, Gastrointestinal bleed, Submucosal tumor of the stomach

## Abstract

**Introduction:**

Gastric lipomas, though rare, are noteworthy for their potential to cause upper gastrointestinal bleeding. This case report highlights the diagnostic and management challenges associated with this uncommon entity.

**Case presentation:**

We present a case of a 39-year-old male with epigastric pain and hematemesis, ultimately diagnosed with a gastric lipoma in the antrum. Endoscopy revealed a submucosal lesion with mucosal ulceration, confirmed by imaging studies. Surgical resection was performed, leading to a favorable outcome.

**Clinical discussion:**

Gastric lipomas are benign tumors that can present with gastrointestinal bleeding, abdominal pain, or obstruction. Diagnosis relies on imaging and endoscopic findings, with biopsy often inconclusive due to the submucosal location. Surgical resection is the preferred treatment for symptomatic or large lipomas, while observation may suffice for asymptomatic lesions.

**Conclusion:**

Gastric lipomas, although infrequent, pose a challenge for diagnosis, especially when associated with bleeding. Surgical resection remains the cornerstone of management for symptomatic or large lipomas, with observation being an option for asymptomatic lesions.

## Highlights

• Gastric lipomas are uncommon mesenchymal tumors, constituting less than 1 % of all gastric tumors.
• A gastric lipoma is a rare tumor that can cause bleeding.
• The management of asymptomatic gastric lipomas can be performed only by observation.
• Traditionally management of symptomatic gastric lipomas is endoscopic resection in small lesions.

## 1 Introduction

Gastric lipomas are uncommon mesenchymal tumors, comprising less than 1 % of all gastric tumors. They are predominantly located in the antrum (1), and may present with gastrointestinal bleeding, abdominal pain, or obstruction ([2], [3], [4]).

Benign in nature, gastric lipomas originate from the submucosal layer and are often discovered incidentally. However, larger tumors can manifest symptoms such as bleeding or obstruction, leading to abdominal pain and, in some cases, intussusceptions (1,3). Diagnosis can be challenging due to their submucosal location and nonspecific symptoms. This case report aims to elucidate the diagnostic and management strategies for gastric lipomas.

We present a case of an unusual cause of gastrointestinal bleeding in a patient who presented with haematemesis, and the condition was addressed through gastrectomy. This work adheres to the SCARE 2023 criteria (5).

## 2 Case report

A 39-year-old man was admitted with a one-week history of epigastric pain accompanied by upper gastrointestinal bleeding, as indicated by hematemesis. The patient had no significant medicalor surgical past history, allergies, or prior drug use. He was a smoker and did not consume alcohol. Physical examination revealed no abnormalities, and per rectal examination did not show any melena. Laboratory tests revealed low hemoglobin levels (6.3 g/dL). The chemistry profile was within normal ranges. The patient received a transfusion, and hemoglobin levels corrected to 10 g/dL.

**Fig. 1:**
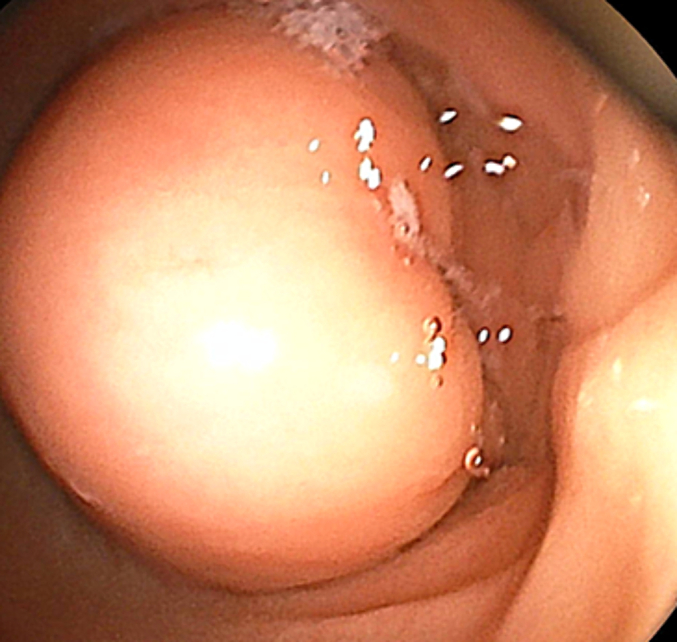
Contrast-enhanced CT scan of the abdomen showed a non-enhancing fat-containing lesion in the antropyloric region of the somach**.**

**Fig. 2:**
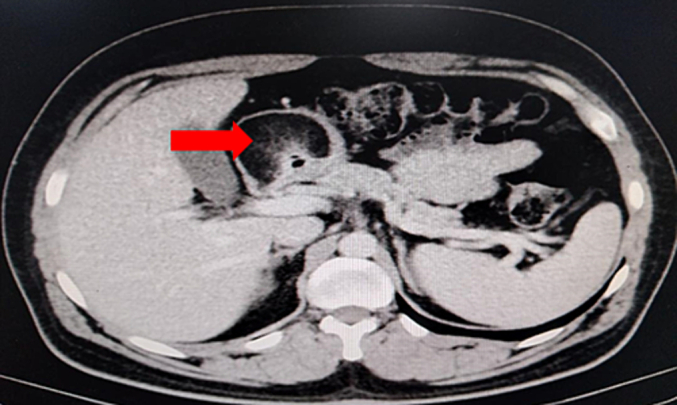
Ulcerated Submucosal tumor.

Upper gastrointestinal endoscopy identified a submucosal lesion in the antrum of the stomach with ulceration of the overlying mucosa (Fig. 1). Biopsy results indicated the absence of malignant signs. Abdominal CT scan displayed a well-defined, heterogeneous, pre-pyloric formation with fat density measuring 6*5 cm in the antropyloric region of the stomach (Fig. 2).

**Fig. 3:**
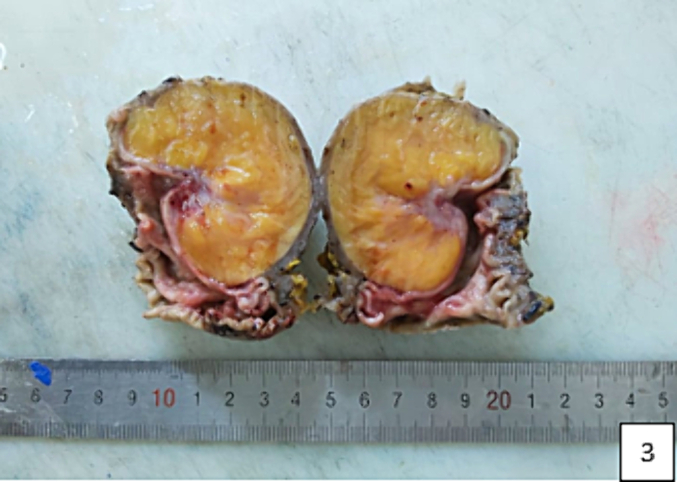
The submucosal tumor is a yellow smooth lobulated lesion concluding to a lipoma tumor. (For interpretation of the references to colour in this figure legend, the reader is referred to the web version of this article.)

**Fig. 4:**
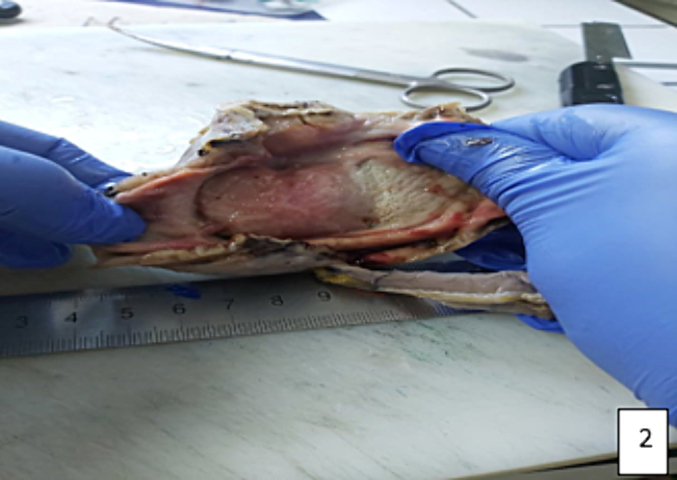
The macroscopic findings of the specimen.

Considering the possibility of a complicated gastric tumor, the patient was evaluated for surgery and subsequently underwent open laparotomy. Intraoperative exploration revealed an antral tumor measuring 5*5 cm. A distal gastrectomy with gastrojejunal anastomosis was performed. The specimen exhibited a smooth submucosal yellow lobulated mass consistent with a lipoma (Figs. 3–4), and histological examination confirmed the diagnosis of a gastric lipoma. The patient reported satisfaction with the intervention, and the postoperative course was uneventful.

## 3 Discussion

Gastric lipomas are uncommon mesenchymal tumors, constituting less than 1 % of all gastric tumors (6,7). They typically manifest in individuals during their fifth or sixth decade of life, with an equal incidence between genders (1,6). In the stomach, these tumors are predominantly located in the antrum, accounting for up to 75 % of cases, and are often situated in a submucosal plane (up to 90 %) (6,7). The majority of gastric lipomas are characterized by small dimensions, ranging from 4 to 9 cm (7). However, in larger tumors, distinctive features may emerge, including a propensity for ulceration, necrosis, inflammation, cystic degeneration, and, infrequently, calcific changes (7).

Gastric lipomas, though rare, pose diagnostic and management challenges due to their nonspecific clinical presentation and submucosal location. In our case, the patient presented with upper gastrointestinal bleeding, highlighting the potential for significant morbidity associated with these lesions.

The majority of gastrointestinal tract lipomas tend to be asymptomatic, the clinical presentation is variable and largely depends on the tumor's size and location (1,6). They typically manifest as incidental findings or nonspecific symptoms, with gastrointestinal bleeding being a relatively uncommon presentation (7). However, when bleeding occurs, it can result from ulceration, necrosis, or inflammation of the overlying mucosa. This underscores the importance of considering gastric lipomas in the differential diagnosis of upper gastrointestinal bleeding, particularly in the absence of other identifiable causes (8).

Diagnosis is based on imaging studies and endoscopic findings, with biopsy often inconclusive due to the submucosal nature of the lesion (6,7).

Diagnosing gastric lipomas can be challenging. While imaging studies such as computed tomography (CT) can provide valuable information, definitive diagnosis often relies on endoscopic evaluation and biopsy (6).

Endoscopic criteria for identifying gastric lipomas include three specific signs: the tenting sign, cushion sign, and naked fat sign (1,6). However, obtaining adequate tissue samples for biopsy can be difficult due to the submucosal nature of the lesion (1). In our case, endoscopy revealed a submucosal lesion with mucosal ulceration, prompting further evaluation and ultimately leading to the diagnosis of a gastric lipoma.

Furthermore, endoscopic ultrasound-guided fine-needle aspiration (EUS-FNA) plays a crucial role in distinguishing lipomas from other potential diagnoses, such as mesenchymal submucosal tumors of the stomach like GIST, leiomyomas, fibroma, and their malignant variants (1,6).

It's important to note that gastric lipomas themselves do not possess malignant potential. However, exceptionally rare cases of synchronous gastric carcinomas have been described, thought to be coincidental (1).

In the medical literature, the treatment of gastric lipomas remains a subject of debate, contingent on presented symptoms and the risk or development of complications. Surgical resection remains the mainstay of treatment for symptomatic or large gastric lipomas. In our case, the patient underwent distal gastrectomy, resulting in a favorable outcome. However, for asymptomatic lesions or those deemed low risk, observation may be a reasonable approach. The decision to intervene surgically should be guided by the presence of symptoms, the risk of complications, and the patient's overall health status (6).

It is worth noting that while gastric lipomas are typically benign tumors, exceptionally rare cases of synchronous gastric carcinomas have been reported. Therefore, thorough evaluation and histological examination of resected specimens are essential to rule out malignancy (3).

To resume our manuscript highlights that gastric lipomas represent an unusual but important cause of upper gastrointestinal bleeding. Clinicians should maintain a high index of suspicion for these lesions, particularly in patients with nonspecific gastrointestinal symptoms or imaging findings suggestive of a submucosal mass. Prompt diagnosis and appropriate management, whether through surgical resection or observation, are crucial for optimizing patient outcomes.

## 4 Conclusion

Gastric lipomas are an unusual cause of upper gastrointestinal bleeding. Clinicians should consider this diagnosis in patients presenting with nonspecific gastrointestinal symptoms, especially when imaging studies reveal a submucosal mass. Surgical resection is curative for symptomatic or large lipomas, while observation may be appropriate for asymptomatic lesions.

## Consent

Written informed consent was obtained from the patient for publication and any accompanying images. A copy of the written consent is available for review by the Editor-in-Chief of this journal on request.

## Ethical approval

The study was approved by the Ethics Committee of Habib Thameur Hospital of Tunisia: Medical Ethics Committee N° CC20Y37.

## Funding

None.

## Authors contribution

All authors were involved in the researching, writing, and editing of the manuscript.

Meryam Mesbahi/Khedhiri Nizar : managed the patient and wrote the first draft

Yazid Benzarti: wrote the first draft

Ben Maamer Anis: helped in editing and reviewing the paper

All authors read and approved the final version to be published.

## Guarantor

Mouna Cherif

## Research registration number

Not applicable.

## Declaration of competing interest

The authors declare no competing interest.
